# Indirect effects of the SARS CoV-2 pandemic on the prevalence of breastfeeding: Modeling its impact

**DOI:** 10.7705/biomedica.5917

**Published:** 2021-10-15

**Authors:** Álvaro Jácome, Carlos Castañeda-Orjuela, Nayide Barahona

**Affiliations:** 1 INLAMA Network, Bogotá, D.C., Colombia INLAMA Network Bogotá D.C. Colombia; 2 Observatorio Nacional de Salud, Instituto Nacional de Salud, Bogotá, D.C., Colombia Observatorio Nacional de Salud Instituto Nacional de Salud Bogotá D.C. Colombia; 3 INLAMA Network, Cartagena, Colombia INLAMA Network Cartagena Colombia

**Keywords:** Breastfeeding, coronavirus infections, health impact assessment, prevalence, pandemics, lactancia materna, infecciones por coronavirus, prevalencia, evaluación del impacto en la salud, pandemias

## Abstract

**Introduction:**

Breastfeeding has a protective effect against acute respiratory and diarrheal infections. There are psychological and social effects due to physical isolation in the population in the mother-child group.

**Objective:**

To assess the impact on infant mortality due to a decrease in the prevalence of breastfeeding during 2020 due to the physical isolation against the SARS CoV-2 (COVID-19) pandemic in Colombia.

**Materials and methods:**

We used the population attributable risk approach taking into account the prevalence of breastfeeding and its potential decrease associated with the measures of physical isolation and the relative risk (RR) of the association between exclusive breastfeeding and the occurrence of acute infection consequences in the growth (weight for height) of children under the age of five through a mathematical modeling program.

**Results:**

We found an increase of 11.39% in the number of cases of growth arrest in the age group of 6 to 11 months with a 50% decrease in breastfeeding prevalence, as well as an increase in the number of diarrhea cases in children between 1 and 5 months of age from 5% (5.67%) on, and an increased number of deaths in children under 5 years (9.04%) with a 50% decrease in the prevalence of exclusive breastfeeding.

**Conclusions:**

A lower prevalence of breastfeeding has an impact on infant morbidity and mortality in the short and medium-term. As a public health policy, current maternal and childcare strategies must be kept in order to reduce risks in the pediatric population.

## Resumen

**Introducción.:**

La lactancia materna tiene un efecto protector frente a infecciones respiratorias y diarreicas agudas. Hay efectos psicológicos y sociales por el aislamiento físico en la población en el grupo materno-infantil.

**Objetivo.:**

Evaluar el eventual impacto en la mortalidad infantil de la disminución en la prevalencia de la lactancia materna durante el 2020 a causa del aislamiento físico por la pandemia del SARS CoV-2 (COVID-19) en Colombia.

**Materiales y métodos.:**

Se utilizó el enfoque de riesgo atribuible poblacional, teniendo en cuenta la prevalencia de la lactancia materna y su potencial disminución asociada con las medidas de aislamiento físico y el riesgo relativo (RR) de la asociación entre la lactancia materna exclusiva y el efecto de la aparición de infecciones agudas en el crecimiento (peso para la altura) de niños menores de cinco años mediante un programa de modelamiento matemático.

**Resultados.:**

Se registró un aumento del número casos de detención del crecimiento en el grupo etario de 6 a 11 meses de 11,39 % al disminuir en 50 % la prevalencia de la lactancia materna, así como un mayor número de casos por diarrea en los cinco primeros meses a partir del 5 % (5,67 %), y un incremento en el número de muertes en menores de 5 años (9,04 %) al disminuirse en 50 % la prevalencia de la lactancia materna.

**Conclusiones.:**

Se registró un impacto en la morbilidad y la mortalidad infantil a corto y mediano plazo al disminuir la prevalencia en la lactancia materna. Como política pública en salud, deben mantenerse las estrategias actuales de atención materno-infantil para disminuir riesgos en la población infantil.

The report of the epidemic outbreak caused by the new SARS CoV-2 coronavirus in Wuhan (China) in December 2019 brought about a new global experience at the world level with profound social, economic, and health repercussions. The declaration of the pandemic ^1^ by the World Health Organization (WHO) unleashed a global emergency in health services as they struggled to deal with the situation.

In the absence of a specific treatment or vaccines against COVID-19 during the early phases of the pandemic, the epidemiological control measures previously used in health crises such as the Spanish flu pandemic in 1918 ^2^, the SARS outbreak in 2002 ^3^, and the MERS epidemic in 2012 ^4^, were implemented to contain SARS CoV-2 and diminish the effect of direct mortality from COVID-19. However, the world is facing short, mid, and long-term indirect consequences of social and economic impact on health systems, mainly in low- and middle-income countries ^5^ where economic and technical resources have been diverted from other programs to manage and control the situation thus weakening other health strategies, such as maternal and child health prevention and prevalent chronic diseases care ^6^.

As it is well known, breastfeeding is a protective factor against potentially severe communicable infections during early childhood, mainly by reducing acute respiratory and diarrheal infections ^7^ risk. Therefore, its active promotion by governments and communities is a key health strategy. In this sense, an indirect negative effect on the prevalence of breastfeeding may occur as an indirect consequence of social isolation due to the SARS CoV-2 pandemic ^6^.

The physical isolation due to SARS CoV-2 in Colombia extended for 156 days from March 25 to August 30, 2020. As of November 13, 2020, Colombia has reported 1,198,746 COVID 19 cases, 1,104,956 recoveries, and 34.031 deaths ^8^. Epidemiological reports have focused mainly on the notification and monitoring of the pandemic ignoring the direct and indirect health impacts in the country.

Different psychological and social effects have been described in the general population subjected to physical isolation for prolonged periods increasing the vulnerability of specific segments. The odds of exposure to violence and physical and mental abuse under these circumstances are higher in the mother-child and elderly groups ^9^^,^^10^. Breastfeeding is crucial in women’s emotional state and stress results in changes in the normal milk production and the mother-child bonding ^11^^-^^13^. Social networks and support groups are important for breastfeeding maintenance ^14^ so limited physical contact during the pandemic ^15^ may have a negative impact on it ^16^. In this context, it is possible to predict a 36.67% decrease in the prevalence of exclusive breastfeeding in our region ^17^. As of 2015, however, there are no published updated data on breastfeeding in Colombia.

The use of mathematical modeling to simulate and predict the potential effects of such a situation on the target population is helpful for evaluating the various scenarios or outcomes. For this effect, the Avenir Health Foundation at Harvard University and the Bill and Melinda Gates Foundation developed the Spectrum program ^18^ to evaluate the impact of changes in the coverage of different health programs. One of its tools, the Lives Saved Tool (LiST), is a mathematical modeling program that has been used for almost 20 years to simulate possible outcomes in maternal and child health ^19^ and is ideal to estimate the impact of variations in breastfeeding prevalence on infant mortality as a result of physical isolation due to the SARS CoV-2 (COVID-19) pandemic during 2020. 

## Materials and methods

**Figure 1:**
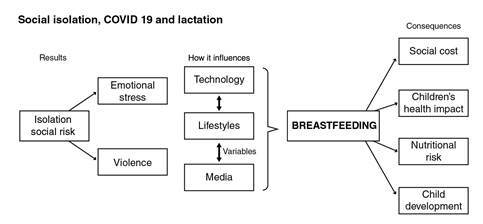
Concept map of breastfeeding and isolation due to COVID-19

We used the LiST tool in the framework of the conceptual model developed for this study to estimate the infant deaths likely to occur as a result of a lower prevalence of breastfeeding in Colombia during 2020 due to changes in the social patterns brought about by the social isolation during the SARS CoV-2 pandemic (figure 1).

We applied the population attributable risk approach in the analysis taking into account breastfeeding prevalence and its potential decrease associated with physical isolation and calculated the relative risk (RR) of the association between breastfeeding and the occurrence of acute infectious diseases, among them diarrheal disease and respiratory infections, and its consequences on the growth (weight for height) of children under five years of age (table 1).

**Table 1:** Mean epidemiological association (relative risks) between breastfeeding prevalence and different health outcomes in children under five years of age according to the mathematical model used

	1 to 5 m	6 to 11 m	12 to 23 m	24 to 59 m
	RR	RR	RR	RR
Diarrhea				
Exclusive breastfeeding	1	1	1	1
Predominant breastfeeding	2.28	1	1	1
Partial lactation	4.62	1	1	1
No breastfeeding	10.52	1.47	2.57	1
Pneumonia				
Exclusive breastfeeding	1	1	1	1
Predominant breastfeeding	1.66	1	1	1
Partial lactation	2.5	1	1	1
No breastfeeding	14.97	1.92	1.92	1
Meningitis				
Exclusive breastfeeding	1	1	1	1
Predominant breastfeeding	1.48	1	1	1
Partial lactation	2.84	1	1	1
No breastfeeding	14.4	3.69	3.69	1
Measles				
Exclusive breastfeeding	1	1	1	1
Predominant breastfeeding	1.48	1	1	1
Partial lactation	2.84	1	1	1
No breastfeeding	14.4	3.69	3.69	1
Whooping cough				
Exclusive breastfeeding	1	1	1	1
Predominant breastfeeding	1.48	1	1	1
Partial lactation	2.84	1	1	1
No breastfeeding	14.4	3.69	3.69	1

Taken from the LiST tool

We defined 2019 as the baseline year and we used the LiST demographic data for Colombia provided by the World Population Prospects 2017 ^20^ for the calculations. We also took into account the exclusive, predominant, and partial breastfeeding parameters reported in the National Nutritional Situation Survey (ENSIN) 2015 ^17^ and the UNICEF projections for the country ^21^ while the baseline infant mortality rates were retrieved from the DANE (*Departamento Administrativo Nacional de Estadística*) databases (table 2).

**Table 2:** Epidemiological and demographic parameters for evaluating the impact of decreased breastfeeding, Colombia, 2020

Demographic data	Value	Year	Source	Observations
Full-term newborn with low birth weight	3%	2018	SIVIGILA*	https://www.ins.gov.co/buscador-eventos/Informesdeevento/ Bajo%20Peso%20al%20Nacer_2018.pdf
Incidence of acute diarrheal disease under five years	140 per 100,000 children under 5 years of age	2019	SIVIGILA	Sivigila: https://www.ins.gov.co/buscador-eventos/ Informesdeevento/ENFERMEDAD%20DIARREICA%20 AGUDA%20PE%20XIII%202019.pdf
Incidence of pneumonia under five years	3.9 per 100,000 children 1 to 4 years old	2017	SIVIGILA	Sivigila: https://www.ins.gov.co/buscador-eventos/ Informesdeevento/Informe%20IRA%20Final%202017.pdf
Infant mortality (deaths less than 1/1,000 live births)	10.7	2017	Vital statistics	https://www.minsalud.gov.co/sites/rid/Lists/BibliotecaDigital/ RIDE/VS/ED/PSP/asis-2019-colombia.pdf
Prevalence of breastfeeding in children under six months			ENSIN 2015 (17)	
Exclusive	36.1% (95%CI 31.1% - 40.4%)	2015		
Predominant (breastfeeding + non-dairy liquids)	3.3% (95%CI 2.3% - 4.8%)	2015		
Predominant (breastfeeding + water)	14.1% (95%CI 11.5% - 17.0%)	2015		
Partial (breastfeeding + formula)	28.3% (95%CI 24.9% - 32.0%)	2015		
No breastfeeding	10.8% (95%CI 8.4% - 13.7%)	2015		
Late neonatal mortality (under 6.9 28 d/1,000 no breastfeeding) in mothers under 18 years of age	6.9	2015	ENSIN 2015 (17)	
Late neonatal mortality (under 7.0 28 d/1,000 no breastfeeding) in mothers from 18 to 49 years of age	7.0	2015	ENSIN 2015 (17)	
Late neonatal mortality (less than 28 days/1000 live births)	7.0	2015	Vital statistics	https://www.minsalud.gov.co/sites/rid/Lists/BibliotecaDigital/ RIDE/VS/ED/PSP/asis-2019-colombia.pdf
Infant mortality (deaths less than	10.7	2017	Vital statistics	https://www.minsalud.gov.co/sites/rid/Lists/BibliotecaDigital/RIDE/VS/ED/PSP/asis-2019-colombia.pdf

*SIVIGILA: Colombian public health surveillance system

We also calculated possible additional deaths in a scenario of decreased breastfeeding prevalence assuming normality in the provision of preventive maternal-perinatal health services during the COVID-19 pandemic quarantine to show the specific expected impact of a lower breastfeeding prevalence.

### Parameters

We used the most recent epidemiological reports in Colombia provided by the Ministry of Health and Social Protection, DANE, the *Instituto Nacional de Salud*, and the ENSIN 2015 for the model (table 2). The baseline value of exclusive breastfeeding prevalence used in the model’s potential scenarios was taken from the ENSIN 2015 (36.1%; 95% CI: 31.1% - 40.4%) ^17^.

For more adjusted breastfeeding prevalence variation ranges, we conducted a literature search in PubMed, Google Scholar, and Embase for reported values of the impact of the 2002 SARS epidemic, the 2012 MERS outbreak, and the 2017 Ebola virus epidemic in Africa on breastfeeding prevalence ^22^^,^^23^ but we found no specific references on the subject. We, therefore, defined four scenarios as follows: No reduction in breastfeeding prevalence with 36.1%; a slight reduction with 34.29% (5% decrease); a moderate one with 32.49% (decrease of 10%), and a high one with 18.05% (50% decrease) compared to the initial value of the breastfeeding baseline prevalence for Colombia.

The infant mortality values in children under 1 year were taken from the 2017 report provided by the Colombian Ministry of Health (table 2) and the demographic data related to breastfeeding from the ENSIN 2015 ^17^. We kept the WHO classification ^24^ for exclusive breastfeeding, predominant breastfeeding, partial breastfeeding, and no breastfeeding (table 2).

### Data analysis

We used the Spectrum software package ^19^^,^^25^ and its LiST module, which is an IT program to model and project different interventions as they relate to changes in pediatric population survival associated with perinatal and child health. We used the Spectrum data for Colombia updated for 2017 in the DemProj module based on the World Population Prospects 2017 ^20^ and those derived from the National Demographics and Health Survey (ENDS 2015) ^26^.

We projected the infant nutritional status data in LiST using the total number and the percentage by the change in weight loss (weight for height) and the growth arrest (height for age). The values obtained were expressed in terms of relative and absolute differences in cases. The unchanged scenario was established with breastfeeding prevalence values based on the initial 2019 data with no variation for 2020 and then compared with the other scenarios. We then contrasted the figures obtained in each breastfeeding prevalence reduction model for 2020 against the expected no-reduction values for the same year and estimated the relative variation in health outcomes. As for 2021, we kept the demographic data obtained for 2020 as no major changes in maternal and child health conditions could be expected.

This model allowed to estimate the potential impact of breastfeeding prevalence variation on the pediatric population nutrition, the alterations in height for age (stunting), the incidence of pathologies such as acute diarrheal disease, acute bronchopneumonia, measles, meningitis, and whooping cough infections, and the changes in infant expected mortality reported as absolute and relative values compared to the no-variation scenario.

## Results

The mathematical model provided the probable scenario of breastfeeding prevalence variation showing an increase in the growth arrest rate (height for age) for the 6 to 11-month age segment (1.08%) when the decrease in breastfeeding prevalence was 5%. This value increased to 11.39% when using a 50% decrease in breastfeeding prevalence (table 3). The total number of additional cases of growth arrest for 2020 would range from 865 to 8,908 depending on the impact on breastfeeding prevalence.

**Table 3:** New cases of growth arrest in children under five years of age according to variations in the prevalence of breastfeeding, Colombia, 2019-2021, according to the mathematical model used

				Absolute difference		Relative difference	
Baseline scenario	2019	2020	2021	2020	2021	2020	2021
0 to 59 months (all children under five years of age)	483,170	478,697	474,575				
<1 month	6,499	6,427	6,356				
1 to 5 months	32,494	32,135	31,779				
6 to 11 months	35,192	34,804	34,418				
12 to 23 months	114,818	113,662	112,984				
24 to 59 months	294,167	291,669	289,038				
**5% decreased prevalence in lactation**							
0 to 59 months (all children under five years of age)	483,170	479,562	476,220	865	1,645	0.18%	0.35%
<1 month	6,499	6,457	6,385	30	29	0.47%	0.46%
1 to 5 months	32,494	32,376	32,016	241	237	0.75%	0.75%
6 to 11 months	35,192	35,183	34,791	379	373	1.09%	1.08%
12 to 23 months	114,818	113,879	113,832	217	848	0.19%	0.75%
24 to 59 months	294,167	291,668	289,196	-1	158	0.00%	0.05%
**10% decreased prevalence in lactation**							
0 to 59 months (all children under five years of age)	483,170	480,385	477,792	1,688	3,217	0.35%	0.68%
<1 month	6.499	6,482	6,410	55	54	0.86%	0.85%
1 to 5 months	32,494	32,592	32,225	457	446	1.42%	1.40%
6 to 11 months	35,192	35,542	35,141	738	723	2.12%	2.10%
12 to 23 months	114,818	114,100	114,657	438	1,673	0.39%	1.48%
24 to 59 months	294,167	291,668	289,359	-1	321	0.00%	0.11%
**50% decreased prevalence in lactation**							
0 to 59 months (all children under five years of age)	483,170	487,605	491,641	8,908	202,603	1.86%	3.60%
<1 month	6,499	6,726	6,652	299	296	4.65%	4.66%
1 to 5 months	32,494	34,597	34,212	2,462	2,433	7.66%	7.66%
6 to 11 months	35,192	38,765	38,337	3,961	3,919	11.38%	11.39%
12 to 23 months	114,818	115,852	121,800	2,190	8,816	1.93%	7.80%
24 to 59 months	294,167	291,665	290,640	-4	1,602	0.00%	0.55%

Regarding the changes in the incidence of acute infectious diseases related to the variation in the prevalence of breastfeeding, the greatest effect was registered in the number of cases of diarrhea and severe diarrhea (table 4). The greatest impact would occur among infants between 1 and 5 months of age as the projected prevalence of breastfeeding decreases between 2019, 2020, and 2021. We observed slight variations in the 2021 expected values compared to those for 2020. Between 111,975 and 2,376,062 additional cases of diarrhea could be expected with a reduction of 473 or 476 cases of severe pneumonia explained by the higher number of deaths from diarrhea.

**Table 4:** New cases of diarrhea in children under five years of age according to varying breastfeeding prevalence, Colombia, 2019-2021, according to the mathematical model used Incidence of cases by pathology

No change in the prevalence of breastfeeding							
Diarrhea	2019	2020	2021				
0 to 59 months	11,729,409	11,620,724	11,518,150				
<1 month	194,834	192,685	190,550				
1 to 5 months	974,170	963,424	952,749				
6 to 12 months	1,169,004	1,156,108	1,143,299				
12 to 23 months	2,321,968	2,298,595	2,284,882				
24 to 59 months	7,069,432	7,009,912	6,946,671				
Severe diarrhea							
0 to 59 months	227,257	225,246	223,257				
<1 month	3,775	3,733	3,692				
1 to 5 months	18,875	18,666	18,460				
6 to 12 months	22,649	22,400	22,151				
12 to 23 months	44,988	44,535	44,270				
24 to 59 months	136,970	135,911	134,685				
5% decreased prevalence in lactation				Absolute difference		Relative difference	
Diarrhea	2019	2020	2021	2020	2021	2020	2021
0 to 59 months	11,729,409	11,841,384	11,735,584	111,975	6,175	0.95%	0.05%
<1 month	194,834	205,924	203,594	11,090	8,760	5.69%	4.50%
1 to 5 months	974,170	1,029,375	1,017,592	55,205	43,422	5.67%	4.46%
6 to 12 months	1,169,004	1,228,528	1,214,520	59,524	45,516	5.09%	3.89%
12 to 23 months	2,321,968	2,367,656	2,353,295	45,688	31,327	1.97%	1.35%
24 to 59 months	7,069,432	7,009,902	6,946,583	-59,530	-122,849	-0.84%	-1.74%
Severe diarrhea							
0 to 59 months	227,257	229,521	227,470	2264	213	1.00%	0.09%
<1 month	3,775	3,990	3,945	215	170	5.70%	4.50%
1 to 5 months	18,875	19,944	19,716	1069	841	5.66%	4.46%
6 to 12 months	22,649	23,803	23,531	1154	882	5.10%	3.89%
12 to 23 months	44,988	45,873	45,595	885	607	1.97%	1.35%
24 to 59 months	136,970	135,911	134,683	-1059	-2287	-0.77%	-1.67%
10% decreased prevalence in lactation				Absolute difference		Relative difference	
Diarrhea	2019	2020	2021	2020	2021	2020	2021
0 to 59 months	11,729,409	12,062,856	11,953,010	333,447	223,601	2.84%	
<1 month	194,834	217,554	215,143	22,720	20,309	11.66%	10.42%
1 to 5 months	974,170	1,092,248	1,077,322	118,078	103,152	12.12%	10.59%
6 to 12 months	1,169,004	1,303,833	1,288,775	134,829	119,771	11.53%	10.25%
12 to 23 months	2,321,968	2,439,330	2,425,271	117,362	103,303	5.05%	4.45%
24 to 59 months	7,069,432	7,009,892	6,946,499	-59,540	-122,933	-0.84%	-1.74%
Severe diarrhea							
0 to 59 months	227,257	233,812	231,683	6,555	4,426	2.88%	1.95%
<1 month	3,775	4,215	4,168	440	393	11.66%	10.41%
1 to 5 months	18,875	21,162	20,873	2,287	1,998	12.12%	10.59%
6 to 12 months	22,649	25,262	24,970	2,613	2,321	11.54%	10.25%
12 to 23 months	44,988	47,262	46,990	2,274	2,002	5.05%	4.45%
24 to 59 months	136,970	135,911	134,682	-1,059	-2,288	-0.77%	-1.67%
50% decreased prevalence in lactation				Absolute difference		Relative difference	
Diarrhea	2019	2020	2021	2020	2021	2020	2021
0 to 59 months	11,729,409	14,105,471	13,974,162	2,376,062	2,244,753	20.26%	19.14%
<1 month	194,834	344,215	340,487	149,381	145,653	76.67%	74.76%
1 to 5 months	974,170	1,722,418	1,702,554	748,248	728,384	76.81%	74.77%
6 to 12 months	1,169,004	1,987,921	1,967,153	818,917	798,149	70.05%	68.28%
12 to 23 months	2,321,968	3,041,107	3,018,177	719,139	696,209	30.97%	29.98%
24 to 59 months	7,069,432	7,009,810	6,945,790	-59,622	-123,642	-0.84%	-1.75%
Severe diarrhea							
0 to 59 months	227,257	273,388	270,843	46,131	43,586	20.30%	19.18%
<1 month	3,775	6,669	6,597	2,894	2,822	76.66%	74.75%
1 to 5 months	18,875	33,372	32,987	14,497	14,112	76.81%	74.77%
6 to 12 months	22,649	38,516	38,114	15,867	15,465	70.06%	68.28%
12 to 23 months	44,988	58,921	58,477	13,933	13,489	30.97%	29.98%
24 to 59 months	136,970	135,909	134,668	-1,061	-2,302	-0.77%	-1.68%

Regarding infant mortality (table 5), an increase in the total number of infant deaths would be expected as a result of a lower breastfeeding prevalence: A 5% decrease would cause an increase of 112 deaths (2.27%) compared with the no-variation scenario with the figure increasing as breastfeeding prevalence decreased. In the scenario of a maximum impact on breastfeeding with a 50% decrease, 975 additional deaths would occur (11.37% increase).

**Table 5:** Variation in breastfeeding the prevalence and expected deaths in children under five per 1000 live births, Colombia, 2019-2021, according to the mathematical model used

						Absolute difference		Relative difference	
Baseline scenario	2019	2020	2021	Relative Increase	Relative increase	2020	2021	2020	2021
Total 0-59 months	10798	10666	10559	-1.22%	-2.21%				
<1 month	5114	5058	5002	-1.10%	-2.19%				
1-59 months	5683	5624	5576	-1.04%	-1.88%				
Mortality in children under five years (deaths per 1000 live births)	14.7	14.7	14.7	0.00%	0.00%				
5% decrease in breastfeeding prevalence									
Total 0-59 months	10798	10778	10672	1.05%	1.07%	112	113	2.27%	3.28%
<1 month	5114	5103	5046	0.89%	0.88%	45	44	1.98%	3.07%
1-59 months	5683	5675	5625	0.91%	0.88%	51	49	1.95%	2.76%
Mortality in children under five years (deaths per 1000 live births)	14.7	14.87	14.39	1.16%	-2.11%	0.17	-0.31	1.16%	-2.11%
10% decrease in breastfeeding prevalence									
Total 0-59 months	10,798	10,864	10,756	1.86%	1.87%	198	197	3.08%	4.08%
<1 month	5114	5141	5085	1.64%	1.66%	83	83	2.74%	3.85%
1-59 months	5683	5723	5672	1.76%	1.72%	99	96	2.80%	3.60%
Mortality in children under five years (deaths per 1000 live births)	14.7	14.95	14.94	1.70%	1.63%	0.25	0.24	1.70%	1.63%
50% decrease in breastfeeding prevalence									
Total 0-59 months	10798	11641	11526	9.14%	9.16%	975	967	10.36%	11.37%
<1 month	5114	5507	5446	8.88%	8.88%	449	444	9.97%	11.07%
1-59 months	5683	6134	6080	9.07%	9.04%	510	504	10.11%	10.92%
Mortality in children under five years (deaths per 1000 live births)	14.7	16.01	16.01	8.91%	8.91%	1.31	1.31	8.91%	8.91%

## Discussion

The results estimated with the LiST tool showed potential increases in the number of cases of infant growth arrest (height for age), the incidence of acute infectious diseases, and mortality both in infants and in children under five years of age as a consequence of the indirect effects of breastfeeding prevalence decrease during social isolation due to the SARS-CoV19 infection in Colombia.

It was foreseeable that SARS CoV-2 containment would involve a series of changes in public health policies and strategies as institutional, budgetary, and healthcare staff resources poured into its management thus affecting already established strategies such as communicable diseases eradication campaigns and maternal and childcare strategies and altering established control routines with consequences and indirect effects that are yet to be documented.

The impact of the implementation of quarantines and containment measures on the general population health has been demonstrated: during the SARS epidemic in 2002, for example, the demand for hospital consultation concerning other diseases decreased as a consequence of people’s refusal to attend due to the risk of contagion ^3^. Something similar was reported during the 2014-2016 Ebola virus epidemic as the use of essential health services decreased, especially the mother-child health care with a short- and mid-term impact on morbidity and mortality in this population segment even greater than that of the infection itself ^27^. This necessarily implies a series of indirect impacts in the mid and long term which need to be evaluated in advance using tools like the one we employed in our analysis.

During social isolation, reports on the emotional state and the effects on mental health in various populations worldwide have increased ^28^ as secondary reactions to such measures with changes in social interaction patterns and an increased incidence of gender and family violence ^29^. Changes in child development due to altered interactions among adult caregivers have also been demonstrated ^10^ with short- and mid-term effects. Social isolation produces emotional stress and anxiety, consequently affecting adult and child behavior with repercussions on coexistence ^30^^,^^31^, life rhythms, and regular routines, and redefining forms of coexistence in confined conditions ^32^^,^^33^. Additionally, structural changes in family dynamics occur due to the shift in family roles with the arrival of new members ^34^^,^^35^, which, under the current conditions, can increase maternal stress and affect breastfeeding ^11^^,^^12^.

Regarding the social determinants of health, the variation in women’s labor market during the period of confinement in Colombia has been evident. According to a report issued by DANE and the Javeriana University, the variation in the labor gap was 22% higher than for male workers. The highest participation of women in the country’s labor market corresponds to the service segment (hotels, retail, restaurants), precisely the most affected during the pandemic. The report also pointed to a proportional reduction in work from home among women as compared to men thus increasing their vulnerability and the trend for unpaid housework, which would probably decrease their time for breastfeeding ^36^.

Interestingly, from a gender perspective, there was a 15.7% decrease in gender-based violence cases in Colombia as reported in the *Instituto Nacional de Salud* weekly epidemiological bulletin for the 2020 fifth epidemiological period ^37^. Sexual violence against boys, girls, and adolescents was most frequently reported, especially prevalent in girls, associated with a proportional increase of neglect in early childhood accounting for 55.9% of the reports. However, it should be noted that a decrease in reporting during the isolation period is likely to have happened ^38^.

The impact of the potential reduction in breastfeeding prevalence according to the mathematical model of the LiST tool reveals an increase of 7.6 to 11.39% in the risk of stunting among children 1 to 5 and 6 to 11 months, i.e., the most vulnerable age groups dependent on adult caregivers ^39^. Growth impairment can affect individual development with overall consequences for the pediatric and future adult population with an estimated OR of 6.19 (95% CI: 3.31 - 11.56) ^40^ consistent with our results. Such changes in these age groups’ growth patterns also have economic consequences for the productive capacity of a country ^41^^,^^42^, which were not evaluated in this research.

The Global International Report ^43^ estimated a prevalence of 11.9% of arrested growth rate in Colombia for 2019. For the Latin American region, the reported reduction in the prevalence of arrested growth rates went from 16.8% in 2009 to 11% in 2019. In the world, the reduction was 11.1% from 21.3% in 2000 ^44^. These values reflect an improvement in global living conditions in the region associated with a decrease in absolute poverty; however, no data are yet available for this year and in the context of the SARS CoV-2 pandemic.

Along the course of life, breastfeeding has a protective effect against various infectious diseases such as acute diarrheal disease and acute respiratory infection. The proportional decrease in exclusive breastfeeding between 1 and 5 months of age and the resulting increase in no-lactation or mixed lactation result in an increased risk of adverse health outcomes with an estimated relative risk of 10.52 (95% CI: 2.79-39.6) ^7^ affecting infant nutritional status and contributing to increasing infant morbidity and mortality due to these infectious diseases, mainly acute diarrheal disease, as evidenced by the modeling ^45^^,^^46^. According to several ecological and observational studies, the lack of breastfeeding increases the risk of acute diarrheal disease incidence, prevalence, mortality, and hospitalization, especially among infants from 0 to 5 months of age. These additional cases also impact the health system resources required for the care of severe cases mainly thus diverting them from COVID-19 or other events management.

A proportional 29% decrease in the number of acute diarrheal disease cases for week 20 in 2020 was reported in the *Instituto Nacional de Salud* weekly epidemiological bulletin ^47^ as compared to the same period in 2019 with an incidence of 41 cases and five deaths in children under one year of age per 1000 inhabitants. In the modeling exercise, the expected number of deaths due to acute diarrheal disease was 207 for every 1000 children under five years of age.

Our study has limitations. First, being a modeling study, its validity depends on the implemented parameters and on the model structure. In this regard, based on recent literature reviews, we used parameters from official national sources and an internationally validated model using the population attributable risk approach. Second, we assumed a breastfeeding prevalence probable range given that no local evaluations measuring this variation are yet available. Such evaluations will identify the most probable impact scenario among those we present in the present analysis. A third limitation refers to the assessment of the impact of physical isolation on breastfeeding exclusively and other preventive programs could also be affected. Hence, our estimates correspond to a conservative scenario given that the effect in the mid and long term could be more significant.

In conclusion, indirect effects of containment measures against COVID-19 are to be expected with the resulting impact on infant morbidity and mortality in the short and midterms as a consequence of breastfeeding prevalence decrease. It is important to maintain and strengthen current strategies for maternal and childcare, as well as to reinforce mental healthcare processes, focusing not only on medical care but also social strategies aimed at individual and family wellbeing, as well as to maintain and implement promotion strategies for breastfeeding focused on primary health care in the framework of the current pandemic.
